# Novel, rare and common pathogenic variants in the *CFTR* gene screened by high-throughput sequencing technology and predicted by *in silico* tools

**DOI:** 10.1038/s41598-019-42404-6

**Published:** 2019-04-17

**Authors:** Stéphanie Villa-Nova Pereira, José Dirceu Ribeiro, Antônio Fernando Ribeiro, Carmen Sílvia Bertuzzo, Fernando Augusto Lima Marson

**Affiliations:** 1 Department of Medical Genetics and Genomic Medicine, School of Medical Sciences, University of Campinas. Tessália Vieira de Camargo, 126, Barão Geraldo, Cidade Universitária Zeferino Vaz, CEP: 13083-887 Campinas, São Paulo Brazil; 2 Department of Pediatrics, School of Medical Sciences, University of Campinas. Tessália Vieira de Camargo, 126, Barão Geraldo, Cidade Universitária Zeferino Vaz, CEP: 13083-887 Campinas, São Paulo Brazil; 3 Laboratory of Pulmonary Physiology, Center for Pediatrics Investigation, School of Medical Sciences, University of Campinas. Tessália Vieira de Camargo, 126, Barão Geraldo, Cidade Universitária Zeferino Vaz, CEP: 13083-887 Campinas, São Paulo Brazil

**Keywords:** Molecular medicine, Genetic predisposition to disease

## Abstract

Cystic fibrosis (CF) is caused by ~300 pathogenic CFTR variants. The heterogeneity of which, challenges molecular diagnosis and precision medicine approaches in CF. Our objective was to identify CFTR variants through high-throughput sequencing (HTS) and to predict the pathogenicity of novel variants through in 8 silico tools. Two guidelines were followed to deduce the pathogenicity. A total of 169 CF patients had genomic DNA submitted to a Targeted Gene Sequencing and we identified 63 variants (three patients had three variants). The most frequent alleles were: F508del (n = 192), G542* (n = 26), N1303K (n = 11), R1162* and R334W (n = 9). The screened variants were classified as follows: 41 – pathogenic variants [classified as (I) n = 23, (II) n = 6, (III) n = 1, (IV) n = 6, (IV/V) n = 1 and (VI) n = 4]; 14 – variants of uncertain significance; and seven novel variants. To the novel variants we suggested the classification of 6b-16 exon duplication, G646* and 3557delA as Class I. There was concordance among the predictors as likely pathogenic for L935Q, cDNA.5808T>A and I1427I. Also, Y325F presented two discordant results among the predictors. HTS and in silico analysis can identify pathogenic CFTR variants and will open the door to integration of precision medicine into routine clinical practice in the near future.

## Introduction

Cystic fibrosis (CF [OMIM: #219700]) is an autosomal recessive disease, clinically characterized by complex phenotypes^1^. Among the phenotypes of the disease, the leading cause of morbidity and mortality is lung disease, caused by cyclic periods of inflammation and infection – mainly by bacteria, and by the obstruction of the pulmonary parenchyma – accumulation of mucus^2^. In CF, abnormal transport of chloride ions and bicarbonate occurs due to structural and functional alterations in the Cystic Fibrosis Transmembrane Conductance Regulator (*CFTR*) gene^3^.

The quantitative and qualitative alterations in the CFTR account for more than 2,000 variants (~300 pathogenic variants in the CFTR2 database and other rare variants)^4^ described in the *CFTR* gene (OMIM: *602421; Cytogenetic location: 7q31.2), homonymous name and encoder of this protein^5,6^. The classification of *CFTR* variants has been recently revised, and currently comprises phenotypic severity, variant type, effect of the CFTR protein and possibility of precision medicine therapy^7,8^. Briefly, classes I (A and B), II and III cause greater phenotypic severity and worse prognosis; while classes IV, V and VI include variants with residual activity of the CFTR protein, consequently, with better prognosis of the disease^7^. The classification of *CFTR* variants plays an important role in studies on gene/protein structure and function, and it has notably been a pillar supporting the use and applicability of targeted corrective therapies – precision medicine^8–10^.

Lately, screening *CFTR* variants has been crucial for genetic counseling, for greater understanding of CF and its diversity/variability and, possibly, for the use of precision medicine^11^. Accordingly, high-throughput sequencing has represented a major breakthrough in CF diagnosis, due to increased information output during *CFTR* sequencing, enabling quick and efficient genotypic identification (*CFTR* variant) with full gene screening, when associated with the identification of deletions and insertions, for example, using MLPA (multiplex ligation-dependent probe amplification)^12,13^. Thus, high-throughput sequencing is one method to determine *CFTR* variability with the aim at encouraging the use of precision medicine, observing its original description: “*an emerging approach for disease treatment and prevention that takes into account individual variability in genes*, *environment*, *and lifestyle for each person*” (Genetics Home Reference, U.S. National Library of Medicine). Consequently, HTS plays a key role in the implementation of corrective therapies in precision medicine and has an impact on the personal and social prognosis of the disease^9,14^. However, in the case of novel or rare variants determined by high-throughput sequencing, the classification of pathogenicity^11^ and possible inclusion in the described classes of *CFTR* become a challenge. In this context, *in silico* tools are essential, and when used in combination with each other and with other prediction tools, they mutually support the process of classification^12^.

Therefore, in this study, our primary aim was to identify genetic variants in the *CFTR* gene in CF patients in a referral center with the use of high-throughput sequencing; and the secondary aim was to determine the pathogenicity of novel variants, rare variants and variants of uncertain significance in the *CFTR* gene by computational methods in order allow classification and applicability of precision medicine, even in orphan cases.

## Cases Under Study and Methods

### CF patients included in the study and diagnosis

This study included 169 samples of genomic DNA from Brazilian CF patients from an admixed population from São Paulo State – Brazil. Related patients were not enrolled. The patients received the diagnosis of CF prior to inclusion in the study due to the presence of clinical signs/symptoms consistent with the disease and after at least two measurements of sweat chloride value ≥ 60 mmol/L. All patients were attended at the Referral Center of a University Hospital and had equal access to: (i) genetic counseling, (ii) tools for diagnosis and functional analysis of CFTR, (iii) outpatient and therapeutic management, (iv) availability of drugs and (v) psychological support. Sweat tests were performed in outpatient settings^15^. Induction of sweating and sweat collection were performed according to the Gibson-Cooke method (1959)^16^, and chloride concentration was quantified by titrations with mercury nitrate^17–20^.

This study was approved by the Ethics Committee of the University of Campinas (CAAE: 78192216.2.0000.5404; opinion: 2.548.490). All patients aged ≥18 years or minor’s parents/guardians signed an Informed Consent Form prior to the beginning of the study. The study protocol followed the ethical principles of the Declaration of Helsinki (1964) and its subsequent amendments.

### High-throughput sequencing of the *CFTR* gene

#### DNA library preparation

The DNA libraries of the CF patients were built along with the positive and the negative controls, using a TruSeq custom amplicon v3.0, according to the manufacturer’s protocol (#1000000002191v04) (Illumina, San Diego, California, USA – all described reagents were obtained from the company through standard protocol – topics 2.2.1 and 2.2.2). The panel design provided 100% covered for all exons and exon/intron boundaries of the *CFTR* gene except exons 2 and 5 (78% and 26% overage, respectively). The panel design included a total of 56 amplicons with 250 base pairs in length to analyze the exons sequences varying between 88 and 1,807 base pairs in length. The protocol for library preparation and detailed information on the panel are shown as Online Supplement 1.

#### Cluster generation and sequencing of DNA libraries

In the cluster generation and in the sequencing of DNA libraries from CF patients, we used a MiSeq sequencer and the inputs MiSeq Reagent Kit v2 and PE MiSeq Flow Cell.

(i). **cluster generation:** the DNA molecules, in a simple tape, bind to the flow cell surface through complementarity with adapters fixed in the ends. Thus, amplification occurs in these areas through the formation of bridges, until the flow cell is full of copies of the region of interest.
(ii). **sequencing of DNA libraries:** sequencing was carried out using a designed panel as previously described. The quality control is demonstrated by the presence of read depth of at least 100 in all alignments and a pass filter (PF-%) of 94% in the dataset. The data were tabulated in customized sample worksheets (known as sample sheet) and the amplicon-identifying file (known as manifest file).

#### Acquisition of data from the sequencing of DNA libraries

The results were analyzed with BaseSpace Sequence Hub (Illumina) – cloud computing tool developed for collection and analysis of sequencing data. Additionally, BaseSpace hosts commercial versions from other developers, which promotes versatility.

Alignment was performed using the TruSeq Amplicon version 3.0 (Illumina) – available in the virtual environment – with the use of the Smith-Waterman (1981)^21^ algorithm in regions delimited by the custom manifest file. Variant calls and annotations were performed in the Illumina VariantStudio v3.0 (Illumina) (vcf, Variant Call Format). The NCBI Reference Sequence was used to perform the *CFTR* variant annotation [GRCh38.p12 (GCF_000001405.38), Ensembl: ENSG00000001626 and MIM: 602421].

Variants identified as likely pathogenic were visually confirmed in the Integrative Genomics Viewer (IGV) version 2.4 (Broad Institute, Cambridge, Massachusetts, USA), having the Human Genome 19 (hg19)^22,23^ as base genome. All single nucleotide variants, insertion or deletions were yielded by high-throughput sequencing and were confirmed by Sanger sequencing^24^. The protocol used is shown as Online Supplement 1. The copy number variants and huge insertions or deletions were screened by multiplex ligation-dependent probe amplification (MLPA).

### Multiplex ligation-dependent probe amplification (MLPA)

The MLPA analysis is based on relative quantification of the number of copies of each region obtained after the amplification reaction by means of hybridization of labeled probes and different sizes of fragments with the genomic DNA of interest. The process of comparing the amplified DNA with the control regions is made visually after the separation and alignment of fragments by the specific equipment. Complementary use of MLPA, in relation to custom panel and/or Sanger sequencing, enables the identification of deleted and/or duplicated regions of DNA, and thus increases the likelihood of identifying the *CFTR* genotype that causes CF^24^.

### Assessment of pathogenicity of variants on the *CFTR* gene

In this study, the classification of variants´ pathogenicity considered some of the consensus criteria of the American College of Medical Genetics and Genomics and Association for Molecular Pathology^25^.

#### Use of databases and population frequency

The variants have greater evidence of pathogenicity, when described in the specific databases, and are supported by functional analysis of valid biological significance. In general, when related to Mendelian disorders, allele frequency is considered a strong indicator for a benign interpretation, as well as when identified in adult and healthy individuals. Thus, we adopted as a criterion for pathogenicity the report/description of the variant in three genetic databases of CF patients, namely (i) cystic fibrosis mutation database [http://www.genet.sickkids.on.ca] – the database was last updated on April 25, 2011; and (ii) CFTR2 – Clinical and Functional Translation of CFTR [http://cftr2.org] – the database was last updated on December 8, 2017 and included a total of 374 variants annotated as (a) CF-causing: 312; (b) variants of varying clinical consequence: 36; (c) non CF-causing: 13; and (d) variants of unknown significance: 13. All the variants in the CFTR2 database were tested and showed the most up-to-date clinical information and results of functional testing available on individual variant or genotype. (iii) and CFTR-France database, that contains data on more than 800 variants, most of which are rare, reported in about 5,000 French individuals with various phenotypes, including CF and CFTR-Related Disease (CFTR-RD)^26^.

Additionally, for variants of uncertain significance in the databases researched and specific to CF, we consulted the databases (a) ClinVar (https://www.ncbi.nlm.nih.gov/clinvar/) – free access database containing information about the interaction genetic variants with clinical phenotypes, with significance at clinical, research or exclusively literary level; (b) InterVar (http://wintervar.wglab.org/) – bioinformatics tool for clinical interpretation of genetic variants that follows the consensus guidelines of the American College of Medical Genetics and Genomics and Association for Molecular Pathology^25,27^, with the following classification: (i) benign, (ii) likely benign, (iii) uncertain significance, (iv) likely pathogenic and (v) pathogenic. Annotation of frequency was made using several databases including: (i) the genome Aggregation Database (gnomAD); (ii) the Exome Aggregation Consortium (ExAC) v0.3; (iii) NHLBI (National Heart, Lung, and Blood Institute) TOPMed: phase III variation data; and (iv) 1000 Genomes Project (human).

An additional comparison included 609 healthy elderly individuals from ABraOM (Online Archive of Brazilian Mutations and Brazilian genomic variants)^28^, which contains genomic variants, including the *CFTR* variants described in this study. The individuals were selected from a census-based sample in the city of São Paulo.

#### Computational methods (*in silico*)

Predictive methods were selected according to their approach and algorithm to complement one another and provide the best identification of the possible degree of pathogenicity of the identified *CFTR* variants. In this study, the predictors were applied in three distinct groups: (i) variants previously described as pathogenic in order to validate the predictors, (ii) variants of uncertain significance in order to identify the possible association with pathogenicity and as a cause of CF and (iii) variants still not described in the literature with the aim of characterizing the pathogenic potential. Thus, the following predictors were applied in the variants identified in the *CFTR*:

(i) MutationTaster (http://www.mutationtaster.org/) evaluates the pathogenicity of the variant through analysis of evolutionary conservation, changes in splice sites, mRNA and protein structure/function. The result is classified as (a) disease causing, (b) disease causing automatic, (c) polymorphism and (d) polymorphism automatic^29^.
(ii) PolyPhen-2 (Polymorphism Phenotyping v2) (http://genetics.bwh.harvard.edu/pph2/) developed for annotation of missense alterations. The output can be classified as (a) unknown, (b) benign, (c) possibly damaging and (d) probably damaging^30^.
(iii) MutPred-2 (Mutation Prediction 2) (http://mutpred.mutdb.org/index.html) analyzes protein sequence through its amino acids. The output is a numerical score in which values > 0.5 denote pathogenicity; and values > 0.8 reduce the chance of false positives to ≤5%, a probabilistic reflection of the alteration being pathogenic. In addition to the score, the software describes the possible consequences of the alteration for the probability of loss or gain of certain structural and functional properties^31^.
(iv) MutPred-LOF (Loss-of-function) developed to evaluate frameshift and nonsense variants, which are generally associated with the greatest impact on protein, with concomitant high likelihood of pathogenicity. The output amplitude ranges from zero to one – and higher score indicate higher pathogenic potential^32^.
(v) MutPred Splice identifies whether the variants in the exon affect splicing, causing alterations in the mRNA. The prediction is categorized into two groups according to score value: (a) ≥0.6: splice affecting variant; and (ii) <0.6: splice neutral variant^33^.
(vi) Human Splicing Finder version 3.1 (http://www.umd.be/HSF3/) locates alterations, calculates the potential splice sites and determines possible branching points. The software provides four pieces of information, namely (a) predicted alteration, (b) prediction algorithm, (c) position of the cDNA and (d) interpretation^34^.
(vii) SNPeffect 4.0 (http://snpeffect.switchlab.org/menu) evaluates the direct implication of the variants on the protein by means of four predictors: (TANGO) predicts the possibility of alterations in protein aggregation as a result of the variability in hydrophobic activity; (WALTZ) evaluates the propensity to form amyloid due to the interference of the variant with protein folding, therefore showing greater accuracy for morphological analysis; (LIMBO) predictor trained from structural modeling to evaluate the binding site for the Hsp70 chaperone that has activity in protein folding and prevents the formation of aggregates of malformed proteins with exposed hydrophobic sequences; and (FoldX) calculates protein stability through the difference in the free energy of each type (wild and mutant)^35^.
(viii) CADD version 1.4 (Combined Annotation Dependent Depletion) (https://cadd.gs.washington.edu/) integrates the analysis of evolutionary conservation, allelic diversity, variants annotation, functional genomic data, transcription information and causal variants within individual genome sequences. A scaled score greater or equal 10 indicates that these are the 10% most deleterious substitution. A score of greater or equal 20 indicates the 1% most deleterious and so on^36^.

**Figure 1:**
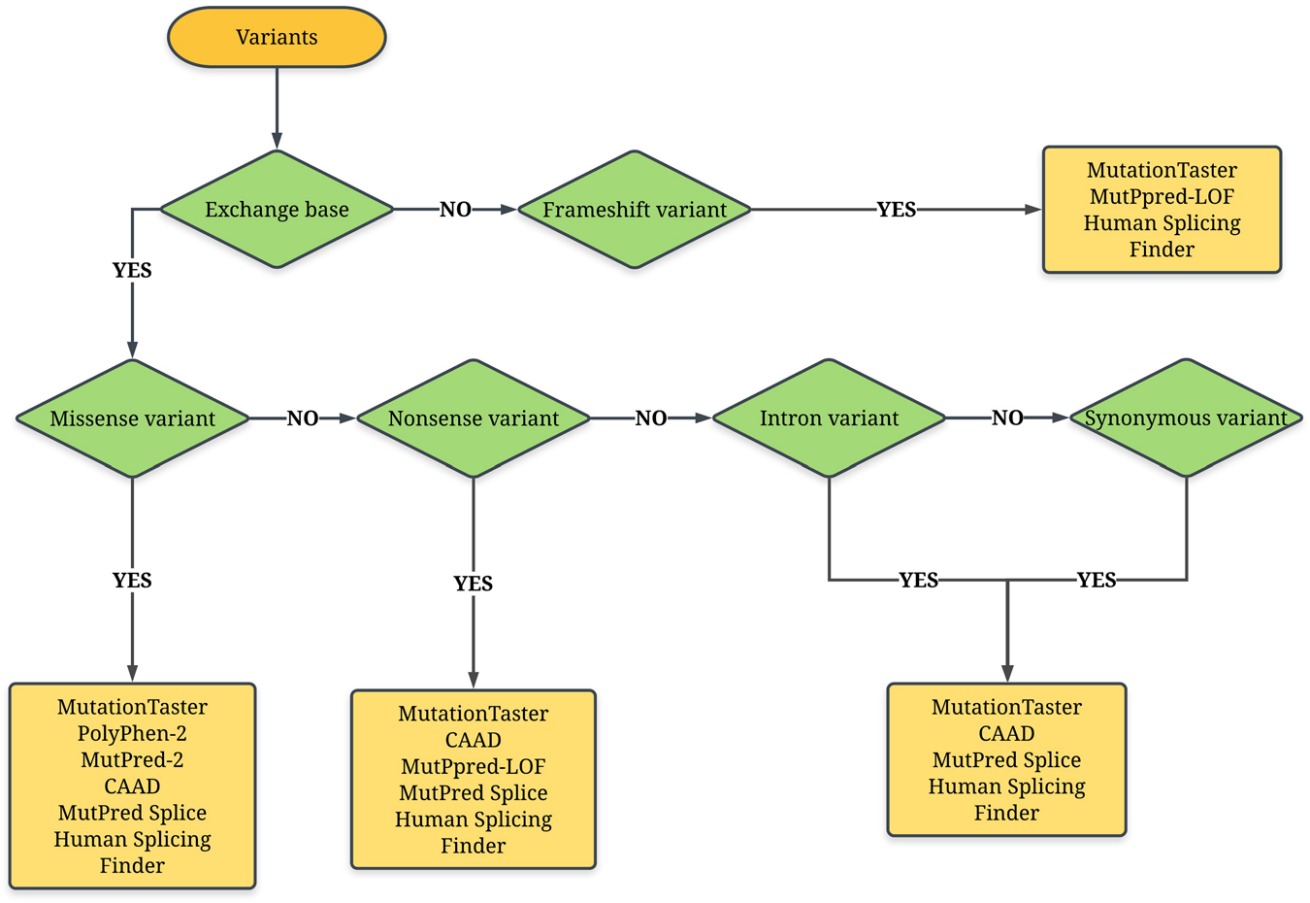
Flowchart showing the steps to characterize the pathogenicity of variants in the *CFTR* gene with the use of predictors: (i) MutationTaster (http://www.mutationtaster.org/); (ii) SNPEffect 4.0 (http://snpeffect.switchlab.org); (iii) PolyPhen-2 (Polymorphism Phenotyping v2) (http://genetics.bwh.harvard.edu/pph2/); (iv) CADD – Combined Annotation Dependent Depletion) (https://cadd.gs.washington.edu/); (v) MutPred-2 (Mutation Prediction 2) (http://mutpred.mutdb.org/index.html); (vi) MutPred-LOF (Loss-of-function); (vii) MutPred Splice; and (viii) Human Splicing Finder version 3.1 (http://www.umd.be/HSF3/). *CFTR*, cystic fibrosis transmembrane conductance regulator.

All predictors can deal with single nucleotide variants, except the MutPred-LOF, which is exclusively used in insertions and deletions. The insertions and deletions can be also analyzed by MutationTaster. The splicing effect was analyzed by the MutPred Splice and Human Splicing Finder. Finally, the flowchart shown in Fig. 1 illustrates the use of the predictors in our study.

### Classification of variants in the *CFTR* gene

The variants in the *CFTR* gene were divided into seven classes according to the following criteria: (a) association with the phenotype of greater severity of the disease, (b) alterations in the DNA, (c) impact on the alteration of the CFTR protein, (d) structural and functional alterations of the CFTR protein and (e) availability and type of drugs available for precision medicine. These are the most recent classification criteria for *CFTR* variants^7–9,37^. The seven classes are listed below:

(i) Class IA: variants that result in the absence of messenger RNA and yet cannot be treated with precision medicine therapy. However, patients with this type of variant may benefit from gene therapy in the future. Examples of Class IA include great deletions and insertions – dele2,3(21 kb).
(ii) Class IB: nonsense variants, which result in the absence of the protein due to degradation of the synthesized immature RNA. In this class, protein correction has been studied using synthetized rescue medications. Examples of Class IB are G542*, W1282*, R553*, Y1092*, G637*, W1282*.
(iii) Class II: variants that result in the absence of the CFTR protein in the cytoplasmic membrane, since errors in processing result in degradation in the endoplasmic reticulum. Thus, drugs that rescue protein trafficking – correcting drugs – have been used. In this class, the most prevalent variant is F508del. Further examples include S549T, A559T, N1303K, I507del, A561E, R1066C.
(iv) Class III: variants that compromise the regulatory site of the protein. Thus, the CFTR protein is found in the plasma membrane and in normal amounts; however, it does not respond to stimulation of cyclic AMP (cAMP), making its opening process not viable. The literature describes the use of drugs that restore CFTR as safe and effective in numerous *CFTR* variants, also known as potentiators. In this class, the following variables can me mentioned: G551D, G551S, G1244E, S1255P, G1349D, S549R.
(v) Class IV: variants that cause reduction in the conduction of chloride ion by the CFTR channel and during the time the channel remains open. In this class, one of the main mechanisms of precision medicine therapy is the use of stabilizing drugs, since the CFTR protein is anchored in the membrane and with residual activity. The following variables can me mentioned: R334W, R347P, A455E, R117H.
(vi) Class V: quantitative reduction in CFTR proteins in the plasma membrane, although these proteins are functional. Numerous mechanisms to correct the problem have been studied, and CF patients appear to present beneficial effect with the use of correctors and potentiators. The following variables can me mentioned: 2789 + 5G > A, 3272-26A > G, 3849 + 10 Kb C > T.
(vii) Class VI: variants that trigger reduction in the stability of the protein, which is degraded rapidly when found in the plasma membrane. Thus, the use of drugs that increase the stability of the protein has been proposed. In this class, the following variables can me mentioned: c.120del23, rF508del (r, rescued).

The NCBI Reference Sequence was used to perform the *CFTR* variant annotation [GRCh38.p12 (GCF_000001405.38), Ensembl: ENSG00000001626 and MIM: 602421].

## Results

A total of 169 patients participated in this study. Molecular analysis of the *CFTR* gene was conducted and the diagnosis was confirmed after the identification of the variants in both alleles. The patients from our referral center had the following characteristics: 46.75% females, 92% Caucasians (self-declared), 91.7% patients with respiratory symptoms, 83.3% patients with digestive symptoms, 15% with meconium ileus, 18.5% with diabetes mellitus, mean age of 16 years and mean age of 91.75 months at diagnosis. Also, regarding the colonization/infection status, the following bacteria were found: *Staphylococcus aureus (*78.5%), *Pseudomonas aeruginosa (*55.8%), mucoid *P*. *aeruginosa* (42%) and *Burkolderia cepacia* (21.85%).

After the end of gene screening in the *CFTR*, a total of 63 variants were identified in the CF patients, and three patients had three variants as follow: (i) c.[1397C > A;3209G > A];[1624G > T]; (ii) c.[1521_1523delCTT];[1000C > T;1241A > C]; (iii) c.[3557delA];[1521_1523delCTT;3140-26A > G]. The cis position was determined by the *CFTR* gene sequencing from the parents of the patients. Moreover, 77 different genotypes were found in our sample, with the highest prevalence observed for the genotypes F508del/F508del, F508del/G542* and F508del/N1303K (p.Asn1303Lys, c.3909C > G), found in 57 (33.73%), 15 (8.88%) and 5 (2.96%) of the patients, respectively. Interestingly, 58/77 (75.32%) of the different genotypes were found in only one CF patient.

In our study, the alleles with higher frequency were: F508del (n = 192; 56.30%), G542* (n = 26; 7.62%), N1303K (n = 11; 3.23%), R1162* (p.Arg1162Ter, c.3484C > T) and R334W (both n = 9; 2.64%). The high prevalence of allele F508del is a CF characteristic, being the most prevalent worldwide. The screened variants were classified as follows: 41 – pathogenic variants [classified according to the alteration in the *CFTR* gene as (I) n = 23 (56.09%), (II) n = 6 (14.63%), (III) n = 1 (2.43%), (IV) n = 6 (14.63%), (IV and V) n = 1 (2.43%) and (VI) n = 4 (9.75%)]; 14 – variants of uncertain significance, considering the findings of the literature and of this study [n = 9 – pathogenic among all predictors (characteristic that gives the high pathogenic potential and association with the diagnosis of CF); n = 5 – discordant among all predictors used]; and seven novel variants.

The novel variants were evaluated and, based on the theoretical type of change + prediction analysis, we recommend that the variants described as duplication of exons 6b-16 [c.(580 + 1_581-1)_(2615 + 1_2616-1)dup], G646* (p.Gly646Ter, c.1936G > T) and 3557delA (p.Gln1186Hisfs*6, c.3557delA) be classified as Class I, and therefore as pathogenic. At the same time, there was agreement between the predictors as likely pathogenic for the variants L935Q (p.Leu935Gln, c.2804T > A), cDNA.5808T > A (c.*1233T > A) and I1427I (p.Ile1427 = , c.4281C > T). In addition, variant Y325F (p.Tyr325Phe, c.974A > T) presented a discordant result between the predictors.

Interestingly, the comparison with the CFF registry data shows that, among the variants previously described as pathogenic, seven variants are not listed in the registry; among the uncertain variants, four are listed. The results are even more divergent when we compare our findings with those of the Brazilian Group of Studies on Cystic Fibrosis. This can be explained due to the lack of information about today’s diversity of *CFTR* variants in our population and/or limited availability of data to the researchers involved in the study on genetics of CF.

**Figure 2:**
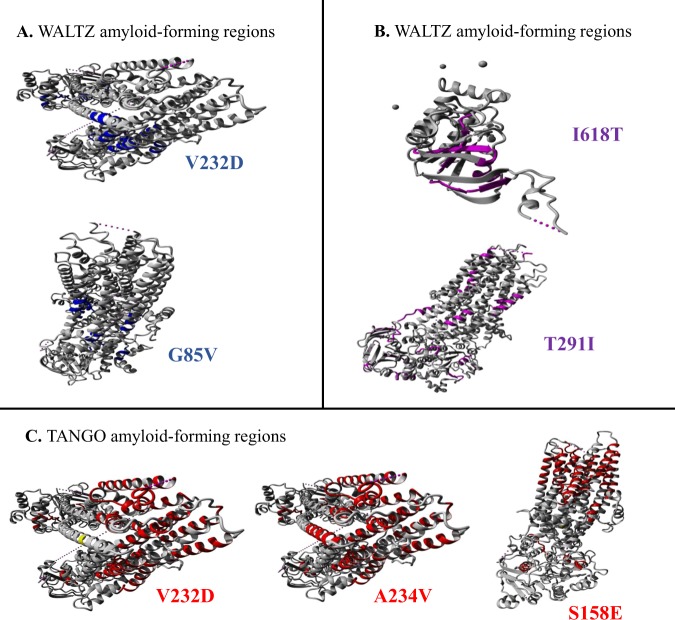
Molecular visualization of WALTZ, LIMBO and TANGO. (**A**) Molecular visualization of WALTZ *amylo*id-forming regions showing the WALTZ aggregation-prone regions as blue-colored segments. (**B**) Molecular visualization of LIMBO chaperone-binding sites showing the LIMBO chaperone-binding sites as pink-colored segments. (**C**) Molecular visualization of TANGO aggregation-prone regions showing the TANGO aggregation-prone regions as red-colored segments. The structural location of the variant residue is colored in yellow. The data was achieved from SNPeffect 4.0 (http://snpeffect.switchlab.org/menu).

**Figure 3:**
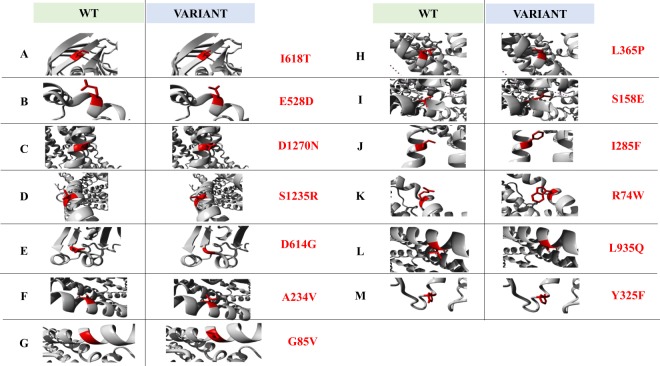
Molecular visualization of the wild-type (WT) (left – red color) and amino acid variant (right – red color) using the FoldX predictor. I, Ile – Isoleucine; T, Thr – Threonine; E, Glu – Glutamic Acid; D, Asp – Aspartate; N, Asn – Asparagine; S, Ser – Serine; R, Arg – Arginine; G, Gly – Glycine; A, Ala – Alanine; V, Val – Valine; L, Leu – Leucine; P, Pro – Proline; F, Phe – Phenylalanine; W, Trp – Tryptophan; Q, Gln – Glutamine; Y, Tyr – Tyrosine. The data was obtained from SNPeffect 4.0 (http://snpeffect.switchlab.org/menu).

**Figure 4:**
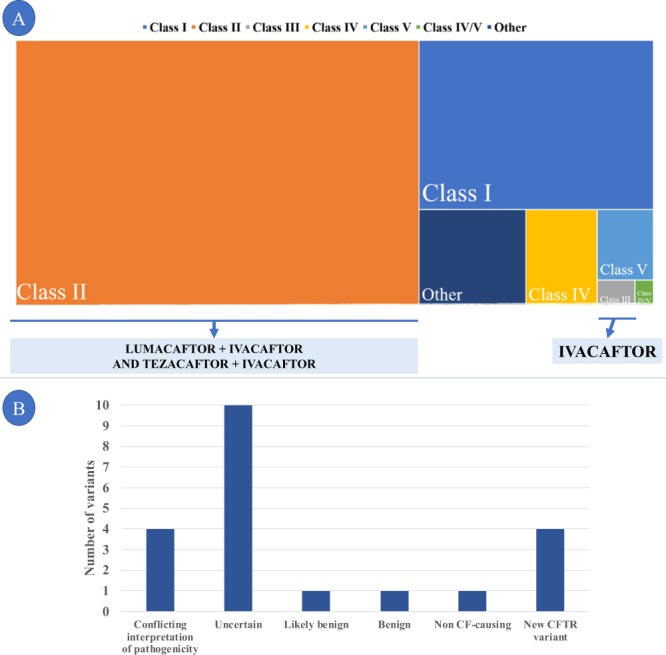
Description of the variants in the *CFTR* gene according to functional classes. (**A**) Map graph (with squares) showing the prevalence of each class of variant in *CFTR* identified in the study sample and the drug, used in precision medicine, available for use with the approval of the FDA (US Food and Drug Administration) – 2018. The approval for use in cystic fibrosis patients is limited to some variants within each class. Therefore, the representation per class is a possibility of grouping the patients and showing, visually, the number of individuals who may have benefits for each class of variant. (**B**) Detailed description of the groups of variants that were not described due to the limitations of the study as belonging to the known classes of *CFTR*, and further studies are needed for the definitive classification. *CFTR*, cystic fibrosis transmembrane conductance regulator. Lumacaftor (CFTR chaperone, VX-809, C_24_H_18_F_2_N_2_O_5_)/ivacaftor (CFTR potentiator, VX-770, C_24_H_28_N_2_O_3_) (Orkambi); Tezacaftor (VX-661, C_26_H_27_F_3_N_2_O_6_) /Ivacaftor (Symdeko); Ivacaftor (Kalydeco).

Briefly, our findings are described in: (i) *CFTR* variants screened in CF patients, considering the allelic analysis, with nomenclature (traditional, c.DNA and protein), n (%), *CFTR* class, dbSNP; prevalence of the variants in the CFTR2 database and in Brazil (according to the registry of the Brazilian Group of Studies on CF), prevalence among CF patients from two studies of Brazil not included in the Brazilian registry and the CFTR-France Database (https://cftr.iurc.montp.inserm.fr/) (Table 1)^26,38,39^; (ii) description of the data of the *in silico* predictors for the variants in the *CFTR* gene not described in the CFF registry, variants with uncertain significance/conflicting as to pathogenicity or novel variants identified in the *CFTR* gene (Table 2 – details of the predictors evaluated in SNPEffect 4.0 are presented in Table 3, Figs 2 and 3); (iii) genotype of patients included in the study, with identification of the two alleles of the *CFTR* gene and 77 different genotypes that were identified (Table 4); and (iv) description of variant classes and therapeutic potential of precision medicine treatments (Fig. 4).

**Table 1:** Full description of *CFTR* variants screened in cystic fibrosis patients considering the alleles.

Traditional name	N	%	cDNA name	Protein name	Predicted functional class	db SNP	CFF (%)^a^	Brazil (%)^b^	São Paulo city (N = 141; %)^c^	Salvador city (N = 50)^d^	CFTR-France Database^e^
**Pathogenic variants in the** ***CFTR*** **gene and previously described in the literature**											
G542*^¥^	26	7.62	c.1624G > T	p.Gly542Ter	IB	rs113993959	2.542	4.32	18 (7.2)	6	161/2019
R1162*	9	2.64	c.3484C > T	p.Arg1162Ter	IB	rs74767530	0.458	1.11	2 (0.8)	3	24/30
2183AA > G	7	2.05	c.2051_2052delAAinsG	p.Lys684SerfsX38	IA	rs121908799	0.382	0.28	2 (0.8)	Not found	38/53
1717-1G > A	3	0.88	c.1585-1G > A	Not applicable	IA	rs76713772	0.856	0.14	1 (0.4)	Not found	76/93
3120 + 1G > A	3	0.88	c.2988 + 1G > A	Not applicable	IA	rs75096551	0.353	1.19	13 (5.2)	1	23/34
S466*^¥^	3	0.88	c.1397C > A	p.Ser466Ter	IB	rs121908805	0.032	Not found	Not found	1	4/4
1812-1G > A	2	0.59	c.1680-1G > A	Not applicable	IA	rs121908794	0.023	0.17	1 (0.4)	Not found	4/4
711 + 1G > T	2	0.59	c.579 + 1G > T	Not applicable	IA	rs77188391	0.193	0.14	1 (0.4)	Not found	29/49
S4*	2	0.59	c.11C > A	p.Ser4Ter	IB	rs397508173	0.01	0.31	6 (2.4)	Not found	3/3
W1282*	2	0.59	c.3846G > A	p.Trp1282Ter	IB	rs77010898	1.215	0.45	Not found	1	55/77
Y1092*	2	0.59	c.3276C > A	p.Tyr1092Ter	IB	rs121908761	0.158	0.20	Not found	Not found	22/30
2184delA	2	0.59	c.2052delA	p.Lys684AsnfsX38	IA	rs121908746	0.18	Not found	1 (0.4)	Not found	9/11
R553*	1	0.29	c.1657C > T	p.Arg553Ter	IB	rs74597325	0.931	0.31	2 (0.8)	Not found	54/76
2556insAT	1	0.29	c.2424_2425dupAT, or c.2421_2422dupAT or c.2422_2423insAT	p.Ser809IlefsX13	IA	rs387906359	0.003	Not found	Not found	Not found	Not found
Y913*	1	0.29	c.2739T > A	p.Tyr913Ter	IB	rs149790377	0.008	Not found	Not found	Not found	0/1
3905insT	1	0.29	c.3773_3774insT	p.Leu1258PhefsX7	IA	rs121908789	0.148	Not found	Not found	Not found	3/4
621 + 1G > T	1	0.29	c.489 + 1G > T	Not applicable	IA	rs78756941	0.931	Not found	Not found	Not found	16/20
Q552*	1	0.29	c.1654C > T	p.Gln552Ter	IB	rs76554633	0.025	Not found	Not found	Not found	Not found
Q890*	1	0.29	c.2668C > T	p.Gln890Ter	IB	rs79633941	0.032	Not found	Not found	Not found	2/2
W1310*	1	0.29	c.3929G > A	p.Trp1310Ter	IB	rs397508645	Not found	Not found	Not found	Not found	Not found
3617delGA	1	0.29	c.3485_3486delGA	p.Val1163LeufsX2	IA	rs397508575	Not found	Not found	Not found	Not found	Not found
622-2A > G	1	0.29	c.490-2A > G	Not applicable	IA	rs397508735	Not found	Not found	Not found	Not found	Not found
1234delGCAAA	1	0.29	c.1234_1238delGCAAA	p.Ala412Thrfs	IA	rs3034796	Not found	Not found	Not found	Not found	Not found
F508del^¥^	192	56.30	c.1521_1523delCTT	p.Phe508del	II	rs113993960	69.744	48.75	147 (59)	11	2,551/3,554
N1303K	11	3.23	c.3909C > G	p.Asn1303Lys	II	rs80034486	1.581	0.94	Not found	1	118/165
A561E	7	2.05	c.1682C > A	p.Ala561Glu	II	rs121909047	0.011	0.06	2 (0.8)	Not found	1/1
R1066C	5	1.47	c.3196C > T	p.Arg1066Cys	II	rs78194216	0.155	0.23	5 (2)	Not found	13/18
G85E	2	0.59	c.254G > A	p.Gly85Glu	II	rs75961395	0.434	0.97	3 (1.2)	Not found	27/34
V232D	1	0.29	c.695T > A	p.Val232Asp	II	rs397508783	Not found	Not found	Not found	Not found	3/9
L206W	1	0.29	c.617T > G	p.Leu206Trp	II	rs121908752	0.023	0.24	1 (0.4)	Not found	24/103
S549R (T > G)	2	0.59	c.1647T > G	p.Ser549Arg	III	rs121909005	0.065	0.40	8 (3.2)	Not found	4/4
R334W^¥^	9	2.64	c.1000C > T	p.Arg334Trp	IV	rs121909011	0.302	1.22	2 (0.8)	3	27/34
D110H	1	0.29	c.328G > C	p.Asp110His	IV	rs113993958	0.046	Not found	Not found	Not found	5/11
D1152H	1	0.29	c.3454G > C	p.Asp1152His	IV	rs75541969	0.402	0.17	1 (0.4)	1	32/101
I618T	1	0.29	c.1853T > C	p.Ile618Thr	IV	rs139468767	Not found	Not found	Not found	Not found	Not found
P205S	1	0.29	c.613C > T	p.Pro205Ser	IV	rs121908803	0.023	0.11	1 (0.4)	Not found	5/8
R347P	1	0.29	c.1040G > C	p.Arg347Pro	IV	rs77932196	0.375	0.09	Not found	Not found	27/35
R1070Q^¥^	1	0.29	c.3209G > A	p.Arg1070Gln	IV/V	rs78769542	0.015	Not found	Not found	Not found	1/1
1716 + 18672A > G	2	0.59	c.1585-9412A > G	Not applicable	V	rs397508229	Not found	Not found	Not found	Not found	2/2
2752-26A > G	2	0.59	c.2620-26A > G	Not applicable	V	rs201716473	0.006	Not found	Not found	Not found	3/4
2789 + 5G > A	2	0.59	c.2657 + 5G > A	Not applicable	V	rs80224560	0.723	0.14	Not found	Not found	66/119
3272-26A > G^¥^	2	0.59	c.3140-26A > G	Not applicable	V	rs76151804	0.331	0.09	Not found	1	30/55
**Variants in the** ***CFTR*** **gene with uncertain/benign/conflicting interpretations of pathogenicity**											
E528D	2	0.59	c.1584G > T	p.Glu528Asp	Conflicting interpretations of pathogenicity*	rs1800095	Not found	Not found	Not found	Not found	Not found
D1270N	1	0.29	c.3808G > A	p.Asp1270Asn		rs11971167	0.039	Not found	Not found	Not found	2/50
S1235R	1	0.29	c.3705T > G	p.Ser1235Arg		rs34911792	0.076	Not found	Not found	Not found	5/48
Q1100P	3	0.88	c.3299A > C	p.Gln1100Pro	Uncertain*	rs397508535	Not found	Not found Not found	Not found	3	Not found
D614G	1	0.29	c.1841A > G	p.Asp614Gly		rs201124247	0.012		Not found	Not found	0/4
A234V	1	0.29	c.701C > T	p.Ala234Val		rs769016520	Not found	Not found	Not found	Not found	Not found
T291I	1	0.29	c.872C > T	p.Thr291Ile		rs779120165	Not found	Not found	Not found	Not found	Not found
G85V	1	0.29	c.254G > T	p.Gly85Val		rs75961395	Not found	Not found	2 (0.8)	Not found	2/3
L365P	1	0.29	c.1094T > C	p.Leu365Pro		rs76727851	Not found	Not found	Not found	Not found	Not found
Q414P^¥^	1	0.29	c.1241A > C	p.Gln414Pro		rs758289310	Not found	Not found	Not found	Not found	Not found
S158R	1	0.29	c.472A > C	p.Ser158Arg		rs397508724	Not found	Not found	Not found	Not found	0/1
I285F	1	0.29	c.853A > T	p.Ile285Phe	Likely benign*	rs151073129	Not found	Not found	Not found	Not found	Not found
A455A	1	0.29	c.1365G > A	p.Ala455=	Benign*	rs79074685	Not found	Not found	Not found	Not found	Not found
R74W	1	0.29	c.220C > T	p.Arg74Trp	Non-CF-causing**	rs115545701	0.025	Not found	Not found	Not found	2/58
**Novel variants screened in the** ***CFTR*** **gene**											
6b-16 exon duplication	1	0.29	c.(580 + 1_581-1)_(2615 + 1_2616-1)dup	Not applicable	IA	Not described	Not described	Not described	Not found	Not found	Not found
G646*	1	0.29	c.1936G > T	p.Gly646Ter	IB	Not described	Not described	Not described	2 (0.8)	Not found	Not found
3557delA^¥^	1	0.29	c.3557delA	p.Gln1186Hisfs*6	IA	Not described	Not described	Not described	Not found	Not found	Not found
L935Q	1	0.29	c.2804T > A	p.Leu935Gln	Not described	Not described	Not described	Not described	Not found	Not found	Not found
cDNA.5808T > A (3′UTR)	1	0.29	c.*1233T > A	Not applicable	Not described	Not described	Not described	Not described	Not found	Not found	Not found
Y325F	1	0.29	c.974A > T	p.Tyr325Phe	Not described	Not described	Not described	Not described	Not found	Not found	Not found
I1427I	1	0.29	c.4281C > T	p.Ile1427=	Not described^f^	Not described	Not described	Not described	Not found	Not found	Not found

N, number of alleles; %, percentage; dbSNP, Single Nucleotide Polymorphism database; CFF, Cystic Fibrosis Foundation; UTR, untranslated region; *CFTR*, cystic fibrosis transmembrane conductance regulator. ^a^Based on the current CFTR2 database (8 December 2017) with 89,052 included patients, and 374 annotated variants: 312 CF-causing; 36 varying clinical consequence; 13 non-CF-causing; 13 unknown significance); ^b^based on the Brazilian Cystic Fibrosis Registry (REBRAFC) with 1,760 patients included; ^c^based on the study entitled as “A new insight into *CFTR* allele frequency in Brazil through next generation sequencing^38^; ^d^based on the study entitled as “Cystic fibrosis: Identification and frequency of mutations in a mixed population from a low-income region in Northeastern Brazil”^39^; ^e^based on list of current CFTR-France Database (https://cftr.iurc.montp.inserm.fr/) – the data is shown as number of alleles in CF patients by the number of alleles in total population; ^f^the variant was not previously detected and more studies should be carried out, but we believe this variant is not a CF-causing variant. *the predicted functional class was not achieved in the CF databases and we included the information regarding the ClinVar (https://www.ncbi.nlm.nih.gov/clinvar/) and InterVar (http://wintervar.wglab.org/); **the variant is described as non-CF-causing in the CFTR2 database. We enrolled 169 cystic fibrosis patients, but three patients showed three variants (^¥^[G542*];[R1070Q;S466*] and [F508del];[R334W;Q414P] and [3557delA];[F508del;3272-26A > G]) in the screening; in this context, the allele frequency was calculated based on 341 alleles.

**Table 2:** Description of *CFTR* variants without inclusion in the CFTR2^a^ database or in Brazilian Cystic Fibrosis Registry^b^, or with uncertain/benign/conflicting interpretations of pathogenicity, or novel variants screened in the *CFTR* gene considering the *in-silico* predictors.

Traditional name	MutationTaster	PolyPhen-2	SNPEffect 4.0	MutPred-2	CADD Phred	MutPred-LOF	MutPred Splice	Human Splicing Finder
**Pathogenic variants in the** ***CFTR*** **gene and previously described in the literature**								
W1310*	Disease causing	Not applicable	Not applicable	Not applicable	42	0.782	Splice affecting variant	New acceptor site; new ESS site; ESE site broken
3617delGA	Disease causing	Not applicable	Not applicable	Not applicable	Not applicable	0.788	Not applicable	ESE site broken
622-2A > G	Disease causing	Not applicable	Not applicable	Not applicable	28.2	Not applicable	Not applicable	Broken WT acceptor Site
1234delGCAAA	Disease causing	Not applicable	Not applicable	Not applicable	Not applicable	0.795	Not applicable	ESE site broken
V232D	Disease causing	Possibly damaging	Alters aggregation and amyloid formation	0.758	23.2	Not applicable	Splice neutral variant	New ESS Site
I618T	Disease causing (no splice change)	Probably damaging	Alters protein stability	0.676	24.7	Not applicable	Splice neutral variant	No significant splicing motif
1716 + 18672A > G	Polymorphism + splice site change	Not applicable	Not applicable	Not applicable	0.81	Not applicable	Not applicable	New donor site
**Variants in the** ***CFTR*** **gene with uncertain/benign/conflicting interpretations of pathogenicity**								
E528D	Disease causing	Benign	Slightly alters protein stability	0.319	23.5	Not applicable	Splice affecting variant	Broken WT donor site; new ESS site
D1270N	Disease causing	Probably damaging	Alters protein stability	0.862	26.4	Not applicable	Splice neutral variant	No significant splicing motif
S1235R	Disease causing	Benign	Alters protein stability	0.524	22.2	Not applicable	Splice neutral variant	No significant splicing motif
Q1100P	Disease causing	Probably damaging	Alters protein aggregation	0.886	23.2	Not applicable	Splice neutral variant	ESE site broken
D614G	Disease causing	Probably damaging	Alters protein stability	0.855	28.2	Not applicable	Splice neutral variant	ESE site broken
A234V	Disease causing	Benign	Alters protein aggregation	0.308	18.8	Not applicable	Splice neutral variant	No significant splicing motif
T291I	Disease causing	Benign	Alters chaperone binding	0.254	17.25	Not applicable	Splice affecting variant	ESE site broken
G85V	Disease causing	Probably damaging	Alters amyloid and severely alters stability	0.902	25.4	Not applicable	Splice affecting variant	New donor site; ESE site broken
L365P	Disease causing	Possibly damaging	Severely alters stability	0.618	25.3	Not applicable	Splice neutral variant	New ESS site
Q414P	Disease causing	Benign	No parameters affected	0.534	23.6	Not applicable	Splice neutral variant	ESE site broken
S158R	Disease causing	Probably damaging	Severely alters stability	0.634	27.2	Not applicable	Splice neutral variant	No significant splicing motif
I285F	Disease causing	Probably damaging	Alters protein stability	0.834	27.2	Not applicable	Splice affecting variant	New donor site; ESE site broken
A455A	Disease causing (no splice change)	Not applicable	Not applicable	Not applicable	5.85	Not applicable	Splice neutral variant	New acceptor site
R74W	Disease causing (no splice change)	Probably damaging	Alters protein stability	0.752	23.3	Not applicable	Splice neutral variant	New ESS site; ESE site broken
**Novel variants screened in the** ***CFTR*** **gene**								
6b-16 exon duplication	Not applicable	Not applicable	Not applicable	Not applicable	Not applicable	Not applicable	Not applicable	Not applicable
G646*	Disease causing (no splice change)	Probably damaging	Not applicable	Not applicable	40	0.792	Not applicable	New donor site; new ESS site
3557delA	Disease causing (no splice change)	Not applicable	Not applicable	Not applicable	Not applicable	0.788	Not applicable	ESE site broken
L935Q	Disease causing (no splice change)	Probably damaging	Alters protein stability	0.841	26	Not applicable	Splice neutral variant	New donor site; ESE site broken
cDNA.5808T > A (ere built along with the positive and the ne)	Disease causing	Not applicable	Not applicable	Not applicable	15.2	Not applicable	Not applicable	Not applicable
Y325F	Disease causing	Benign	Alters protein stability	0.236	22.2	Not applicable	Splice neutral variant	ESE site broken
I1427I	Disease causing (no splice change)	Not applicable	Not applicable	Not applicable	9.856	Not applicable	Splice neutral variant	ESE site broken

UTR, untranslated region; *CFTR*, cystic fibrosis transmembrane conductance regulator; ESE, exonic splicing enhancer; ESS, exonic splicing silencer; WT, wild-type. ^a^Based on the current CFTR2 database (8 December 2017) with 89,052 patients included, and 374 annotated variants: 312 CF-causing; 36 varying clinical consequence; 13 non-CF-causing; 13 unknown significance); ^b^based on the Brazilian Cystic Fibrosis Registry (REBRAFC) with 1,760 included patients. (i) MutationTaster (http://www.mutationtaster.org/); (ii) SNPEffect 4.0 (http://snpeffect.switchlab.org); (iii) PolyPhen-2 (Polymorphism Phenotyping v2) (http://genetics.bwh.harvard.edu/pph2/); (iv) CADD – Combined Annotation Dependent Depletion) (https://cadd.gs.washington.edu/); (v) MutPred-2 (Mutation Prediction 2) (http://mutpred.mutdb.org/index.html); (vi) MutPred-LOF (Loss-of-function); (vii) MutPred Splice; (viii) Human Splicing Finder version 3.1 (http://www.umd.be/HSF3/).

**Table 3:** Description of *CFTR* variants without inclusion in CFTR2^a^ database or in the Brazilian Cystic Fibrosis Registry^b^, or with uncertain/benign/conflicting interpretations of pathogenicity, or novel variants screened in the *CFTR* gene considering the SNPEffect 4.0 predictors.

Traditional name	dTANGO	dWALTZ	dLIMBO	dFoldX
**Pathogenic Variants in the** ***CFTR*** **gene and previously described in the literature**				
V232D	Decreases the aggregation tendency	Increases the amyloid propensity	Does not affect the chaperone binding tendency	No effect on the protein stability
I618T	Does not affect the aggregation tendency	Does not affect the amyloid propensity	Increases the chaperone binding tendency	Reduces the protein stability
**Variants in the** ***CFTR*** **gene with uncertain/benign/conflicting interpretations of pathogenicity**				
E528D	Does not affect the aggregation tendency	Does not affect the amyloid propensity	Does not affect the chaperone binding tendency	Slightly reduces the protein stability
D1270N	Does not affect the aggregation tendency	Does not affect the amyloid propensity	Does not affect the chaperone binding tendency	Slightly reduces the protein stability
S1235R	Does not affect the aggregation tendency	Does not affect the amyloid propensity	Does not affect the chaperone binding tendency	Slightly reduces the protein stability
Q1100P	Increases the aggregation tendency	Does not affect the amyloid propensity	Does not affect the chaperone binding tendency	—
D614G	Does not affect the aggregation tendency	Does not affect the amyloid propensity	Does not affect the chaperone binding tendency	Reduces the protein stability
A234V	Decreases the aggregation tendency	Does not affect the amyloid propensity	Does not affect the chaperone binding tendency	Enhances the protein stability
T291I	Does not affect the aggregation tendency	Does not affect the amyloid propensity	Increases the chaperone binding tendency	No effect on the protein stability
G85V	Does not affect the aggregation tendency	Decreases the amyloid propensity	Does not affect the chaperone binding tendency	Severely reduces the protein stability
L365P	Does not affect the aggregation tendency	Does not affect the amyloid propensity	Does not affect the chaperone binding tendency	Severely reduces the protein stability
Q414P	Does not affect the aggregation tendency	Does not affect the amyloid propensity	Does not affect the chaperone binding tendency	—
S158R	Decreases the aggregation tendency	Does not affect the amyloid propensity	Does not affect the chaperone binding tendency	Reduces the protein stability
I285F	Does not affect the aggregation tendency	Does not affect the amyloid propensity	Does not affect the chaperone binding tendency	Reduces the protein stability
R74W	Does not affect the aggregation tendency	Does not affect the amyloid propensity	Does not affect the chaperone binding tendency	Enhances the protein stability
**Novel variants screened in the** ***CFTR*** **gene**				
L935Q	Does not affect the aggregation tendency	Does not affect the amyloid propensity	Does not affect the chaperone binding tendency	Reduces the protein stability
Y325F	Does not affect the aggregation tendency	Does not affect the amyloid propensity	Does not affect the chaperone binding tendency	Slightly reduces the protein stability

*CFTR*, cystic fibrosis transmembrane conductance regulator. ^a^Based on the current CFTR2 database (8 December 2017) with 89,052 included patients, and 374 annotated variants: 312 CF-causing; 36 varying clinical consequence; 13 non-CF-causing; 13 unknown significance); ^b^based on the Brazilian Cystic Fibrosis Registry (REBRAFC) with 1,760 included patients. (i) SNPEffect 4.0 (http://snpeffect.switchlab.org).

**Table 4:** *CFTR* genotype from cystic fibrosis patients and drugs approved by the FDA^a^.

Genotype	N	%	Drug	Genotype	N	%	Drug
F508del/F508del	57	33.73	Orkambi or Symdeko	F508del/L935Q	1	0.59	Orkambi
F508del/G542*	15	8.88	Orkambi	F508del/I1427I	1	0.59	Orkambi
F508del/N1303K	5	2.96	Orkambi	F508del/A234V	1	0.59	Orkambi
F508del/2183AA > G	4	2.37	Orkambi	F508del/T291I	1	0.59	Orkambi
F508del/R1162*	4	2.37	Orkambi	F508del/D1152H	1	0.59	Orkambi/Kalydeco
F508del/1717-1G > A	3	1.78	Orkambi	F508del/6b-16 exon duplication	1	0.59	Orkambi
F508del/1716 + 18672A > G	2	1.18	Orkambi	F508del/G646*	1	0.59	Orkambi
F508del/1812-1G > A	2	1.18	Orkambi	F508del/G85V	1	0.59	Orkambi
F508del/2789 + 5G > A	2	1.18	Orkambi	F508del/L365P	1	0.59	Orkambi
F508del/A561E	2	1.18	Orkambi	F508del/P205S	1	0.59	Orkambi
F508del/G85E	2	1.18	Orkambi	F508del/Q552*	1	0.59	Orkambi
F508del/Q1100P	2	1.18	Orkambi	F508del/Q890*	1	0.59	Orkambi
F508del/R1066C	3	1.78	Orkambi	F508del/R334W	1	0.59	Orkambi
F508del/Y1092*	2	1.18	Orkambi	[F508del];[R334W;Q414P]	1	0.59	Orkambi
G542*/N1303K	2	1.18	Not applicable	F508del/R347P	1	0.59	Orkambi
R334W/G542*	2	1.18	Not applicable	F508del/R553*	1	0.59	Orkambi
R334W/R334W	2	1.18	Not applicable	F508del/S1235R	1	0.59	Orkambi
2183AA > G/2183AA > G	1	0.59	Not applicable	F508del/S158E	1	0.59	Orkambi
2183AA > G/N1303K	1	0.59	Not applicable	F508del/S466*	1	0.59	Orkambi
2752-26A > G/2752-26A > G	1	0.59	Not applicable	F508del/S4*	1	0.59	Orkambi
3120 + 1G > A/R1066C	1	0.59	Not applicable	F508del/S549R	1	0.59	Orkambi/Kalydeco
3120 + 1G > A/R1162*	1	0.59	Not applicable	F508del/W1310*	1	0.59	Orkambi
3617delGA/3905insT	1	0.59	Not applicable	G542*/2556insAT	1	0.59	Not applicable
622-2A > G/711 + 1G > T	1	0.59	Not applicable	G542*/A561E	1	0.59	Not applicable
A561E/A561E	1	0.59	Not applicable	G542*/I618T	1	0.59	Not applicable
A561E/Y913*	1	0.59	Not applicable	G542*/Q1100P	1	0.59	Not applicable
E528D/E528D	1	0.59	Not applicable	G542*/R1162*	1	0.59	Not applicable
Y325F/cDNA.5808T > A	1	0.59	Not applicable	G542*/S549R	1	0.59	Kalydeco
D110H/V232D	1	0.59	Kalydeco	I285F/A455A	1	0.59	Not applicable
D614G/R1162*	1	0.59	Not applicable	L206W/W1282*	1	0.59	Kalydeco
F508del/2184delA	1	0.59	Orkambi	N1303K/N1303K	1	0.59	Not applicable
F508del/2184insA	1	0.59	Orkambi	[G542*];[R1070Q;S466*]	1	0.59	Kalydeco
F508del/3120 + 1G > A	1	0.59	Orkambi	R1162*/R1162*	1	0.59	Not applicable
F508del/3272-26A > G	1	0.59	Orkambi	R334W/R1066C	1	0.59	Not applicable
[3557delA];[F508del;3272-26A > G]	1	0.59	Orkambi	R74W/D1270N	1	0.59	Kalydeco
F508del/621 + 1G > T	1	0.59	Orkambi	S466*/A561E	1	0.59	Not applicable
F508del/711 + 1G > T	1	0.59	Orkambi	S4*/N1303K	1	0.59	Not applicable
F508del/1234delGCAAA	1	0.59	Orkambi				

^A^The traditional nomenclature was used to define the *CFTR* genotype. CFF, Cystic Fibrosis Foundation; N, number of cystic fibrosis patients; %, percentage. The drugs are marked according to the approval of the U.S. Food and Drug Administration (FDA). In this case, we did not consider the *CFTR* class to determine the use of precision medicine.

No alleles related to the novel variants were found in the ABraOM dataset. However, the following pathogenic variants described by allele frequency (n) were observed: (1 allele) R1162*, V232D, D1152H, D1270N, Q1100P, A455A, R74W; (2 alleles) 2184delA; (3 alleles) F508del, I285F; (8 alleles) S1235R; and (33 alleles) E528D.

## Discussion

Precision medicine has played a key role in effective treatment of CF patients. The identification of patients´ genotype, which was a major challenge in the past, has become a major milestone in CF management. Thus, CF is a study model which has provided major therapeutic and scientific advancements in identifying the genotypes of *CFTR*. The implementation of high-throughput sequencing has also proven to be effective with a large amount of generated data. It is also necessary to learn more about the classification of the variants regarding pathogenicity, structure, function and protein activity associated with the use of precision medicine^11^. We identified 63 variants and 77 different genotypes in the 169 CF patients included in the study, and three patients had three variants. Of the variants identified, 41 were identified as pathogenic according to the literature [http://cftr2.org], 14 had uncertain significance [http://cftr2.org] and 7 were previously unknown. The complex allele c.[1397C > A;3209G > A] has been described in a CF patient at the homozygous state in CFTR-France and in many cases at the literature as a severe effect on CF function^26,40,41^. On the other hand, the complex allele c.[1000C > T;1241A > C] was never reported to our knowledge. Note that the c.3140-26A > G has been reported in complex allele with Phe508del after newborn screening^42^.

Following the consensus recommendation of the American College of Medical Genetics and Genomics and Association for Molecular Pathology, the terms “mutation” and “polymorphism”, which have been widely used, were replaced with the term “variant”. In this context, the variants are classified as (i) pathogenic, (ii) likely pathogenic, (iii) uncertain significance, (iv) likely benign and (v) benign^25^. In our study, the classification was based on combined criteria, and the variant was considered of uncertain significance in cases of conflict of interpretation between the criteria. Here, special emphasis was given to conservation, as it has consistently been shown as a key criterion for the identification of pathogenic variants^43–45^.

### Importance of identifying *CFTR* variants

Numerous hypotheses have been proposed in an attempt to answer questions related to the variability of the disease, with basis on the understanding of the relation between the phenotype of the CF and the genotype of the *CFTR* gene. In addition to the environmental and socioeconomic criteria involving patients, genetic composition is the main factor of this characterization, being an important guideline for the prediction, evolution and therapy of the disease^7–9,37,46^.

At the same time, molecular identification allows family groups to better understand the disease and receive the customized genetic counselling^37^. Through molecular diagnosis, CF patients prompt a network of genetic information within the family, which now becomes a risk group for the presence of at least one pathogenic variant. Following the classification of the variants, *in silico* tools have been used in the cases of uncertainty as to the degree of pathogenicity of the variant^12^.

Likewise, genetic identification is necessary due to the development of drugs to correct, enhance and stabilize the CFTR protein in CF^12^. So, the use of precision medicine should be evaluated to include the genotypes and phenotypes of greater prevalence, at least *in vitro* orphan variants and, possibly, variants of unknown characterization.

Thus, in recent years, CF has been a study model for genetic and phenotypic correlation, as well as for the use of precision medicine, contributing significantly to the scientific advances in these areas^8–10^.

### Importance in determining the class of the *CFTR* variants and the severity of the alteration

The classification of variants according to their functional significance in the protein and, more recently, according to the specific corrective treatment predicted by the new drugs is crucial to optimize the therapy and provide information to organize databases. It will further enable universal access to the treatment, considering the genotype characteristics and its expression within a complex environment that is the reflection of the environment and of numerous other genes that influence the outcome of the disease^1,46^.

For example, we can mention the CF drugs that are approved by the FDA (U.S. Food and Drug Administration): (i) Orkambi [Lumacaftor 100 or 200 mg (VX-809, C_24_H_18_F_2_N_2_O_5_) + Ivacaftor 125 mg (VX-770, C_24_H_28_N_2_O_3_)] for patients aged 2 years or older (the European Medicines Agency approved the use of the drug for patients aged 6 years or older) and with the F508del variant, with total annual costs estimated at ~$259,000 for the treatment; (ii) Symdeko [Tezacaftor (VX-661, C_26_H_27_F_3_N_2_O_6_) 100 or 150 mg + Ivacaftor 150 mg] for patients with the F508del/F508del genotype and aged 12 years or older, with total annual costs estimated at $322,560 for the treatment; (iii) Kalydeco (Ivacaftor 150 mg) has approval for use in individuals aged 2 years or older and with at least one copy of the variants belonging to Class III (E56K, G178R, S549R, K1060T, G1244E, P67L, E193K, G551D, A1067T, S1251N, R74W, L206W, G551S, G1069R, S1255P, D110E, R347H, D579G, R1070Q, D1270N, D110H, R352Q, S945L, R1070W, G1349D, R117C, A455E, S977F, F1074L, R117H, S549N, F1052V and D1152H), with total annual costs estimated at $344,100 for the treatment. In our sample, approximately 140 CF patients are eligible for the use of at least one of these drugs, with total annual costs estimated at $40,308,420 for the treatment in only one referral center in Brazil. In this case, the classification of *CFTR* variants was not taken into account, since the FDA did not approve of the use for all variants which are described within a class of the *CFTR* (Table 5). Health is viewed as priceless and it can be evaluated in high economic terms. Health care costs rise exponentially when considering the use of precision medicine. The drug price was established by Vertex Pharmaceutics (Northern Avenue, Boston, MA, U.S.) – https://www.vrtx.com.

**Table 5:** Approved drugs to be used in cystic fibrosis treatment based on precision medicine in one referral center in Brazil.

Drug	N	Monthly cost/patient	Annual cost/patient	Total annual cost
Orkambi or Symdeko^a^	57	$ 26,880	$ 322,560	$ 18,385,920
Orkambi	76	$ 21,583	$ 259,000	$ 19,884,000
Kalydeco	5	$ 28,675	$ 344,100	$ 1,720,500
Orkambi^b^ or Kalydeco	2	$ 21,583	$ 259,000	$ 518,000
Total				$ 40,308,420

^a^The values were based on Symdeko; ^b^the values were based on Orkambi.

### The use of predictors – critical view

The use of predictors (*in silico*) for pathogenicity has surpassed science to become a helpful tool for the genetic diagnosis of several diseases in the clinical practice, including CF^44^. Predictors are considered to have low biological relevance, mainly due to their roots in computational and mathematical algorithms^44^, but when associated with one another and with other methods (clinical analysis, laboratory diagnosis, validation of findings), they are an important step to discover the molecular basis of genetic diseases^25^. In this context, two positive sweat tests in two samples (gold standard) confirmed the diagnosis of CF in our study population. The genetic analysis included the use of predictive tools, which showed concordance with the results obtained from the sweat tests^29–35^.

One variant was identified in the *CFTR* and was submitted to only two predicting tools given the place where it occurred – 3′UTR (cDNA.5808T > A). The MutationTaster was applied to all identified *CFTR* variants as well as Human Splicing Finder and MutPred Splice (analysis of variants that alter the splice site) to almost all *CFTR* variants, considering other variants of uncertain significance, novel or not described variants in the CFTR2 database or registry of the Brazilian Group of Studies on CF. The MutatiosnTaster, Human Splicing Finder and MutPred Splice is broadly used because they support numerous types of input. Finally, PolyPhen-2, MutPred-2, SNPeffect 4.0 and CADD were used to evaluate alterations of amino acids exchange, and an exploratory analysis was performed with MutPred-LOF for nonsense and frameshift variants.

In our study, there was consensus on pathogenicity in at least two predictors among the variants previously cited in the literature as pathogenic [in short W1310*, 3617delGA, 622-2A > G, 1234delGCAAA, V232D, I618T and c.1585-9412A > G] and that were not included in the CFTR2 database or in the registry of the Brazilian Group of Studies on CF.

Following the same line of reasoning, *CFTR* variants considered as of uncertain significance and, therefore with conflicting interpretation as to the degree of pathogenicity, had the evaluation conducted by *in silico* prediction. In all cases, the possible degree of pathogenicity of missense variants was confirmed with mutual comparisons among predictors. Thus, MutationTaster, PolyPhen-2, MutPred-2, SNPeffect 4.0 and CADD, respectively, presented as outcomes: disease causing; probably damaging/possibly damaging; higher values of the score – cutoff = 0.500; affect the aggregation tendency, amyloid propensity, chaperone binding tendency and protein stability; and pathogenic (score greater or equal 10) for the variants: D1270N (p.Asp1270Asn, c.3808G > A), Q1100P (p.Gln1100Pro, c.3299A > C), D614G (p.Asp614Gly, c.1841A > G), G85V (p.Gly85Val, c.254G > T), L365P (c.1094T > C, p.Leu365Pro), S158R (p.Ser158Arg, c.472A > C), I285F (p.Ile285Phe, c.853A > T) and R74W (p.Arg74Trp, c.220C > T). In the analysis of alteration for splice site, the predictors MutationTaster, MutPred Splice and Human Splicing Finder, respectively, presented the following outcomes: disease causing, splice affecting variant and Broken WT donor site; new ESS site or ESE site broken for variants E528D (p.Glu528Asp, c.1584G > T); and T291I (p.Thr291Ile, c.872C > T). On the other hand, for variants Q414P (p.Gln414Pr, c.1241A > C) and A455A (p.Ala455 = , c.1365G > A), MutationTaster presented the result as disease causing, and the Human Splicing Finder yielded ESE site broken and new acceptor site, respectively. Finally, variants S1235R (p.Ser1235Arg, c.3705T > G) and A234V (p.Ala234Val, c.701C > T) presented only the alteration in MutationTaster and CADD as disease causing and pathogenic, respectively.

In our study we highlight the use of predictors for missenses variants should be conservative. Some predictors have a huge sensitivity with a poor specificity and positive predictive value (causing a high false positive rate) when used for some *CFTR* variants prediction. In this group of predictors tools, we can include the MutationTaster and CADD, which may predict as pathogenic some neutral variants (consequence on their interpretation in asymptomatic individuals like partners for example). Moreover, another limitation of predictors is their inability to correlate a “pathogenic” prediction with the phenotypic spectrum of a variant. As an example, S1235R variant is predicted as pathogenic/disease-causing but this does not necessarily “involved in CF”, and this variant should rather be considered CFTR-RD^47^. In this way, we need to emphasize the importance of epidemiological data to refine the phenotypic spectrum of variants.

Thus, the predictors are complementary tools to determine and define the pathogenicity of a variant, and in some cases rely on the interpretation of the findings and validity between different prediction tools. The disparity between some of the findings may be caused by computational limitations arising from the specificity of the numerical algorithms that go against the breadth and complexity of biological mechanisms. Therefore, *in silico* tools of greater robustness and diverse in their neural networks (such as MutPred-2 and SNPeffect 4.0) can present criteria for the best definition of pathogenicity.

### Detailed description of the novel variants

Certain genetic changes are recognized in the literature as having greater impact on proteins. Moreover, the location where they are placed is relevant given the possible alterations in splice sites. Novel variants, in loci already described with other pathogenic alleles, must be carefully studied, because they are considered, in theory, as having prior evidence of pathogenicity.

The novel variants identified in our study were determined and classified according to the theoretical framework for types of alteration associated with the prediction analyses. Hence, G646* (nonsense) and 3557delA (*frameshift*) were identified as Class I. Both variants had consistent results for pathogenicity between the *in-silico* predictors used.

The duplication from exon 6b to exon 16 cannot be submitted to any predictive test as its input is incompatible with the software available. However, according to the theoretical interpretation of the duplication of 10 exons and the intercalating non-coding regions, it was considered as great severity. We also suggest that this variant should belong to Class I. Furthermore, according to the revision of the Human Genome Variation Society (HGVS), the nomenclature c.(580 + 1_581-1)_(2615 + 1_2616-1)dup was proposed for the variant.

Regarding the limitation in use of predictors, we only proposed some hypotheses about the genetic classification of the other novel *CFTR* variants. In this case, the *missense* variant L935Q was considered as possibly pathogenic with six predictions consistent with the outcome of pathogenicity (MutationTaster – disease causing; PolyPhen-2 – probably damaging; MutPred-2 – score of 0.841; SNPeffect 4.0 – reduces the protein stability; CADD – score of 26; Human Splicing Finder – new donor site; and ESSE site broken). On the other hand, variant cDNA.5808T > A in region 3′UTR was submitted to only two predictors (MutationTaster – disease causing and CADD – score of 15.2) that determined it as pathogenic. Finally, synonym variant I1427I and missense variant Y325F were considered as disease causing, reduces the protein stability and ESE site broken, in MutationTaster, SNPeffect 4.0 and Human Splicing Finder respectively. However, in use of CADD, both variants showed an antagonist result, observing the scores of 9.85 and 22.2 for the I1427I and Y325F, respectively. In addition, the variant Y325F was considered as benign in PolyPhen-2.

The novel variants were identified in heterozygosity with F508del in the CF patients, except for variants cDNA.5808T > A and Y325F, which were identified in the same patient, possibly indicating a genotype. In addition, variant 3557delA was identified in a complex genotype, namely c.[3557delA];[1521_1523delCTT;3140-26A > G]. A factor to be considered in our study is the analysis of splicing, which was applied in almost all variants and yielded numerous positive results related to the mechanisms involved in this type of alteration, including variants not located in the consensus region. Also, in cases of splicing, the interpretation that supports the theory stating that use of the predictor should be or not associated with prior knowledge of the variant location, pathogenic potential and protein expression studies, considering the specificity of each case^48^. In example, the I1234V which is a true splicing mutation and has no impact as an amino acid change was evaluated and was not adequately predicted by existing in silico prediction models^49^. To prove and to confirm the I1234V (c.3700A > G; p.Ile1234Val) action an *in vitro* approach was in native tissues from patients, emphasizing the relevance of functionally characterizing unclassified variants *ex vivo* and/or *in vitro* for disease diagnosis, prognosis and for therapy assessment.

### Study limitations

(i) despite the use of custom gene panels, some regions of the *CFTR* gene were difficult to cover (exon 2 and exon 5), which is considered a technical limitation in the detection of variants;
(ii) the scientific literature is still controversial about the applicability of predictive tools to predict the pathogenicity in case of novel variants, mainly missense ones;
(iii) prediction tools can compile information from different sources, such as analysis of evolutionary conservation, position of the variant in the genome and formation and function of the protein; but in some predictors, there may be a misinterpretation regarding the pathogenicity of the variants. And yet, the interaction of numerous methods and pieces of information, including the use of clinical and laboratory characteristics, concomitantly with the use of predictors and identified variants, can lead to the correct and viable use of *in silico* pathogenicity prediction.

### Highlights

(i) a high number of variants (63) were identified in the *CFTR* gene;
(ii) 68 unique genotypes were found in the CF patients;
(iii) seven novel variants were identified in the *CFTR* gene, which represents an update of 6/2,049 (0.0029%) in the *CFTR* database only in one study after thirty years of the *CFTR* gene identification;
(iv) 14/63 variants in the *CFTR* gene were characterized as of uncertain or conflicting significance regarding pathogenicity;
(v) *in silico* predictors showed viability and reliability in the analysis of *CFTR* variants.

### Perspective

The techniques available for molecular analysis applied to medical genetics represent a major advance in the CF diagnosis and encourage a new therapeutic approach, which includes the treatment of symptoms and, in many cases, of the disease itself. This therapeutic approach requires a combination of factors, such as funding pharmacological research, fostering research and expanding acquired laboratory knowledge to medical practice, which are an interdisciplinary approach. As a result of the varying genetic background of CF patients, many rare variants may cause CF, which complicates the management of the disease using precision medicine therapy. However, a patient with a rare and/or orphan variant may in the future be included in individual clinical trials, and this will be possible if the *CFTR* genotype is first identified, followed by the classification of the variant and *in vitro* clinical trials. In addition, precision medicine moves forward in CF management with corrective, enhancing and stabilizing drugs (with temporary action) for therapies that act directly in the correction of the genetic problem, including gene therapy and gene-editing techniques. An example is the use of CRISPR-Cas9 – Clustered Regularly Interspaced Short Palindromic Repeats-associated protein 9). In this context, CF is a study model in precision medicine^50,51^.

## Conclusion

High-throughput sequencing has entirely reshaped molecular diagnosis of CF, and custom panels have proved to be effective in detecting rare and novel variants of the *CFTR* gene. Furthermore, our sample showed the high diversity in the variants identified, even in a small geographic area, as they occurred in isolation in approximately ¾ of the CF patients. In addition, the use of predictors is an important step for the classification of pathogenicity, especially of variants of uncertain significance, rare and/or novel variants, being showed in our study the viability and reliability of these tools.

## Supplementary information

Methods
