# Supplementary material for: Novel, rare and common pathogenic variants in the CFTR gene screened by high-throughput sequencing technology and predicted by in silico tools
Source: Sci Rep. 2019 Apr 17;9:6234. doi: 10.1038/s41598-019-42404-6 (PMC6470152; doi:10.1038/s41598-019-42404-6)
Supplement: Supplementary file 1 — Methods [file 41598_2019_42404_MOESM1_ESM.pdf]

Authors: Stephanie Villa-Nova Pereira, José Dirceu Ribeiro, Antônio Fernando Ribeiro, Carmen Sílvia Bertuzzo, Fernando Augusto Lima Marson

METHODS

PART I. High-throughput sequencing of the *CFTR* gene

DNA library preparation

The DNA libraries of the CF patients were built, along with the positive and the negative control, in compliance with the protocol (#1000000002191v04) available by the supplier (Illumina, San Diego, California, USA – all described reagents were obtained from the company through standard protocol – topics 2.2.1 and 2.2.2). The complete protocol is shown as **Online supplement**, including the following phases:

(i) preparation of the input: the DNA samples were quantified by fluorimetry in Qubit 2.0 (Life Technologies, Carlsbad, California, USA) with the use of Qubit dsDNA HS (high sensitivity) assay kit (Q32851, Life Technologies), diluted in H_2O at a concentration of 10 ng/µL. The samples were quantified again for preparation of the input of 10 ng in four µL of resuspension solution 1 (2.5 ng/µL). After this procedure, one µL of sample stabilization solution 1 was added.

(ii) hybridization of oligonucleotide: hybridization of the oligonucleotides pool (upstream and downstream) specific to the regions of interest was performed in Veriti 96-Well Thermal Cycler (Applied Biosystems, Waltham, Massachusetts, USA).

(iii) removal of the oligonucleotides in suspension: the separation and removal of the oligonucleotides in suspension from the other components of the solution were performed with magnetic beads with genomic DNA affinity. Removal of oligonucleotides represents a critical step in building libraries due to the possible loss of DNA, since the beads are sensitive to temperature and dryness and may loosen the DNA that will be removed along with the oligonucleotides in suspension.

(iv) oligonucleotide extension and ligation: DNA polymerase replicates the DNA sequence from the upstream oligonucleotide (3' end) toward the region of interest, limited by the downstream oligonucleotide (5' end) by DNA ligase. The reaction product contains the regions of interest flanked by the sequences necessary for amplification.

(v) amplification of DNA libraries: each sample was identified by the combination of indexes (i7 and i5) incorporated into the amplification sequences. The indexes cannot be contaminated and must represent a single combination/sample. Amplification was performed in 29 cycles, according to the number of amplicons, following the manufacturer's protocol.

(vi) DNA library clean-up: similar to step (iii), with the use of magnetic beads to separate PCR products from other components in solution during the reaction. After removal, the product was evaluated in 4% agarose gel and submitted to electrophoresis to identify the amplified fragments of ~350 bp.

(vii) DNA library normalization: the libraries were normalized to a similar concentration for all samples in order to reduce the likelihood of non-homogeneous sequencing among the products. This process occurred through beads that bind to DNA until reaching saturation. This is a critical step as it relies on the use of beads and is subject to handling errors.

(viii) pool of DNA libraries: the libraries (in equal volume) were combined in a single tube.

(ix) denaturation and dilution of DNA libraries: 15 μL of the DNA library pool were diluted in 585 μL of hybridization buffer 1, in agreement with the Illumina technical support. Denaturation of the DNA library pool occurs by incubation on a plate preheated to 98°C for two minutes, followed by five minutes at zero degrees – with subsequent application on the flow cell.

<u>PART II.</u> CUSTOM PANEL – Reference genome: GRCh37.p13

Target	Start Coordinate	Stop Coordinate	Length	Amplicons	Coverage
**CFTR_Exon_17062085**	117251610	117251887	278	2	100
**CFTR_Exon_17062412**	117231963	117232736	774	4	100
**CFTR_Exon_17062882**	117174305	117174444	140	1	26
**CFTR_Exon_17063273**	117144282	117144442	161	1	78
**CFTR_Exon_17063779**	117180129	117180425	297	2	100
**CFTR_Exon_17064325**	117304717	117304939	223	2	100
**CFTR_Exon_17064491**	117267551	117267849	299	2	100
**CFTR_Exon_17065287**	117175277	117175490	214	2	100
**CFTR_Exon_17065648**	117170928	117171193	266	2	100
**CFTR_ThreeUtrExon_17065718**	117306937	117308743	1807	12	100
**CFTR_Exon_17065837**	117282467	117282672	206	2	100
**CFTR_Exon_17066201**	117182045	117182187	143	1	100
**CFTR_Exon_17066583**	117254642	117254792	151	1	100
**CFTR_Exon_17067608**	117230382	117230518	137	1	100
**CFTR_Exon_17062219**	117176577	117176752	176	2	100
**CFTR_Exon_17062567**	117243561	117243861	301	2	100
**CFTR_Exon_17062979**	117305488	117305643	156	1	100
**CFTR_Exon_17063554**	117246703	117246832	130	2	100
**CFTR_Exon_17063795**	117250548	117250748	201	2	100
**CFTR_Exon_17064341**	117199493	117199734	242	2	100
**CFTR_Exon_17064795**	117227768	117227912	145	1	100
**CFTR_Exon_17065407**	117234959	117235137	179	1	100
**CFTR_Exon_17065694**	117292871	117293010	140	2	100
**CFTR_FiveUtrExon_17065760**	117119992	117120226	235	2	100
**CFTR_Exon_17065902**	117149063	117149221	159	1	100
**CFTR_Exon_17066466**	117242855	117242942	88	1	100
**CFTR_Exon_17067074**	117188670	117188902	233	2	100

<u>Part III.</u> Evaluation of the variants in the *CFTR* gene by Sanger sequencing

The *CFTR* gene is composed of 27 exons; but as exon 13 is large in size, it was amplified in 2 fragments (13.1 and 13.2). The amplification conditions of each of the 28 fragments were optimized for the final volume of 50 µL containing 50 ng of DNA, 10 picomoles of each primer, 1.5 U of Taq, final concentrations of 1.5 mM of $MgCl_2$, 80 µM of each deoxyribonucleotide, 1X buffer with KCl [50mM KCl, 10 mM Tris-HCl pH 8.4, 0.08% (v/v) Nonidet P40] or 1X buffer with $(NH_4)_2SO_4$ [20 mM $(NH_4)_2SO_4$; 75 mM Tris-HCl pH 8.4; 0.01% (v/v) Tween 20]. The reactions were carried out in parallel with a control DNA sample.

The primer sequence, annealing temperatures, buffer size and amplified fragments are described in **Table 1** of the **Online supplement**. *CFTR* exon sequencing, including exon/intron boundaries, was performed as previously described.

Fragment	Primers (5'- 3')	Buffer	T – annealing	bp
**1**	S: CGT AGT GGG TGG AGA AAG C<br>AS: CCT TTA CCC CAA ACC CAA CC	$(NH_4)_2SO_4$*	64.6	392
**2**	S: ATT CCA AAT CTG TAT GGA GAC C<br>AS: GTT GGG ATT ACA GGC ATT AGC	KCl	60.5	315
**3**	S: AGA TAT CTG GCT GAG TGT TT<br>AS: TGT GAT ACA TAA TGA ATG TAC	KCl	56	318
**4**	S: TGT GTT GAA ATT CTC AGG GT<br>AS: TTG TAC CAG CTC ACT ACC T	KCl	61	376
**5**	S: ATT TCT GCC TAG ATG CTG GG<br>AS: AAC TCC GCC TTT CCA GTT GT	KCl	64	395
**6a**	S: TTA GTG TGC TCA GAA CCA CG<br>AS: CTA TGC ATA GAG CAG TCC TG	KCl	56	386
**6b**	S: TGG AAT GAG TCT GTA CAG CG<br>AS: GAG GTG GAA GTC TAC CAT GA	KCl	63	415
**7**	S: AGA CCA TGC TCA GAT CTT CCA T<br>AS: GCA AAG TTC ATT AGA ACT GAT C	KCl	60.5	410
**8**	S: TGA ATC CTA GTG CTT GGC AA<br>AS: TCG CCA TTA GGA TGA AAT CC	KCl	55	359
**9**	S: GTC CTC TAG AAA CCG TAT GC<br>AS: ACT ACA CCC ATA CAT TCT CC	$(NH_4)_2SO_4$*	65	500
**10**	S: GCA GAG TAC CTG AAA CAG GA<br>AS: CAT TCA CAG TAG CTT ACC CA	KCl	61	491
**11**	S: CAA CTG TGG TTA AAG CAA TAG TGT<br>AS: GCA CAG ATT CTG AGT AAC CAT AAT	KCl	60	425
**12**	S: GTG AAT CGA TGT GGT GAC CA<br>AS: CTG GTT TAG CAT GAG GCG GT	KCl	61	426
**13.1**	S: TGC TAA AAT ACG AGA CAT ATT GC<br>AS: ATC TGG TAC TAA GGA CAG	KCl	60.5	528
**13.2**	S: TCA ATC CAA TCA ACT CTA TAC G<br>AS: TAC ACC TTA TCC TAA TCC TAT GAT	KCl	58	498
**14a**	S: AAA AGG TAT GCC ACT GTT AAG<br>AS: GTA TAC ATC CCC AAA CTA TCT	KCl	56	512
**14b**	S: GAC CCA GGA ACA CAA AGC A<br>AS: GTC ACC TCA CCC AAC TAA TG	KCl	59.5	365
**15**	S: CGA TTT TGA GGT TAA GGG TGC<br>AS: AAG GCA CAT GCC TCT GTG CA	KCl	63	483
**16**	S: AAT GCG TCT ACT GTG ATC CA<br>AS: TGT GGG ATT GCC TCA GGT TT	KCl	61	401
**17a**	S: ATC ACT GAC ACA CTT TGT CC<br>AS: CCA AAA TGA AGT CAC ATG GT	KCl	61	440
**17b**	S: ATT CAA AGA ATG GCA CCA GT<br>AS: GAT AAC CTA TAG AAT GCA GC	$(NH_4)_2SO_4$*	51	464
**18**	S: AAT GTG ATA TGT GCC CTA GG<br>AS: ACA GAT ACA CAG TGA CCC TC	KCl	59.5	342
**19**	S: GCC CGA CAA ATA ACC AAG TG<br>AS: GCT AAC ACA TTG CTT CAG GCT	KCl	64	454
**20**	S: GGT CAG GAT TGA AAG TGT GCA<br>AS: CTA TGA GAA AAC TGC ACT GGA	KCl	64	401
**21**	S: AAT GTT CAC AAG GGA CTC CA<br>AS: CAA AAG TAC CTG TTG CTC CA	KCl	59	476
**22**	S: AAA CGC TGA GCC TCA CAA GA<br>AS: TGT CAC CAT GAA GCA GGC AT	KCl	64	565
**23**	S: GTG GCT AAC GCT ATA TCA AC<br>AS: ACA TGG CTC AGA TCA AAG TG	KCl	59.5	436
**24**	S: GGA CAC AGC AGT TAA ATG TG<br>AS: CAT GTC AAC ATT TAT GCT GC	KCl	63	386

Table 1. Conditions for amplification of DNA fragments of the *CFTR* gene - screening for the presence of variants

T, temperature; S, sense primer; AS, antisense primer; bp, base pairs. *, addition of 5% DMSO (Dimethyl sulfoxide or dimethyl sulfoxide) in the reaction.

PART IV. Computational methods (*in silico*) to classify pathogenicity

Predictive methods were selected according to their approach and algorithm, in order to complement one another and provide the best identification of the possible degree of pathogenicity of the identified *CFTR* variants. In this study, the predictors were applied in three distinct groups: (i) variants previously described as pathogenic in order to validate the predictors, (ii) variants of uncertain significance in order to identify the possible association with pathogenicity and as a cause of CF and (iii) variants still not described in the literature with the aim of characterizing the pathogenic potential. Thus, the following predictors were applied in the variants identified in the *CFTR*:

(i) MutationTaster (http://www.mutationtaster.org/) evaluates changes in splice sites, mRNA, protein structure/function and pathogenicity of the variant through analysis of evolutionary conservation. The input is accomplished with the combination of different information, including gene identifier, transcript identifier, sequence type and genetic alteration (base exchange, insertion or deletion) and with the description of a small sequence containing the variant. The result is classified by the Bayesian model that calculates the likelihood of pathogenic potential of the variants as (a) disease causing, (b) disease causing automatic, (c) polymorphism and (d) polymorphism automatic. "Automatic" indicates the presence of previous description in genetic databases. In addition to the above classification, each analysis yields complementary information, such as type of alteration, possible alterations in protein, splice site, regulatory sites and allele frequency previously observed. MutationTaster was validated five times through prediction models and has 91.1 ± 0.1% accuracy; however, its main limitation lies in its inability to analyze insertions and deletions with over 12 bp[29].

(ii) PolyPhen-2 (Polymorphism Phenotyping v2) (http://genetics.bwh.harvard.edu/pph2/) developed for annotation of missense alterations. Using a Bayesian model, the software allows the prediction of the pathogenic potential of the variant in the protein based on the amino acid sequence in FASTA files. It collects data on protein structure and function, alignment of sequence, phylogeny, evolutionary conservation and description in databases. Thus, numerous aspects are evaluated, including characterization of exchange and location in the sequence, likelihood of each allelic variant, mapping of spatial structure of the protein (or homologous) to identify electrostatic alterations or interactions with other ligands. The output can be classified as (a) unknown, (b) benign, (c) possibly damaging and (d) probably damaging[30].

(iii) MutPred-2 (Mutation Prediction 2) (http://mutpred.mutdb.org/index.html) analyzes protein sequence through its amino acids in the FASTA files. The input must contain between 30 to 30,000 amino acids

and the analysis time is proportional to the number of amino acids and substitutions. In MutPred-2, 14 structural and functional properties are evaluated. The output is a numerical score in which values > 0.5 denote pathogenicity, and values > 0.8 reduce the chance of false positives to ≤ 5%, a probabilistic reflection of the alteration being pathogenic. In addition to the score, the software describes the possible consequences of the alteration for the probability of loss or gain of certain structural and functional properties[31].

(iv) MutPred-LOF (Loss-of-function) developed to evaluate frameshift and nonsense variants, which are generally associated with the greatest impact on protein, with concomitant high likelihood of pathogenicity. The predictor makes use of the analysis of a network of factors, including evolutionary conservation, signal peptide, ligation, regulation, structure and dynamics of the protein, enzyme activity, reported homology and posttranslational modifications. MutPred-LOF presents reported performance with area under the Receiver Operating Characteristic (ROC) curve. The output amplitude ranges from zero to one – and higher score indicate higher pathogenic potential[32].

(v) MutPred Splice identifies whether the variants in the exon affect splicing, causing alterations in the mRNA. The prediction is categorized into two groups according to score value: (a) ≥ 0.6: splice affecting variant; and (ii) < 0.6: splice neutral variant[33].

(vi) Human Splicing Finder version 3.1 (http://www.umd.be/HSF3/) locates alterations, calculates the potential splice sites and determines possible branching points. The software provides four pieces of information, namely (a) predicted alteration: determines the type of signal that will be altered by the variant [for example: new cryptic site created (donor or acceptor), altered branching or splice point]; (b) prediction algorithm: introduces the algorithm(s) that predicted the alteration, requiring the use of at least two different algorithms (of six) for the alteration to be considered as relevant; (c) position of the cDNA: graphical representation of the region in which the variant occurs, as well as of the divergence or nondivergence in the data obtained in the predictors used. In the same graph, the wild-type sequence (green – if there was a breakage site) or the altered sequence (red – if a new splice site is created) are described; (d) interpretation: a brief description about the potential biological effect of the predicted signal and its influence on the splice site[34].

(vii) SNPeffect 4.0 (http://snpeffect.switchlab.org/menu) is a 4-preditctor tool that evaluates the direct implication of the variants on the protein through the correlation of numerous algorithms: (TANGO) predicts the possibility of alterations in protein aggregation as a result of the variability in hydrophobic activity; (WALTZ) evaluates the propensity to form amyloid due to interference of the variant with protein folding, therefore showing greater accuracy for morphological analysis; (LIMBO) predictor

trained from structural modeling to evaluate the binding site for the Hsp70 chaperone that has activity in protein folding and prevents the formation of aggregates of malformed proteins with exposed hydrophobic sequences; and (FoldX) calculates protein stability through the difference in the free energy of each type (wild and mutant). Prediction analysis considers the structural and functional sites by identifying catalytic regions, secondary structures, membrane topology and cell processing by the numerous posttranslational modifications[35].

(viii) CADD version 1.4 (Combined Annotation Dependent Depletion) (https://cadd.gs.washington.edu/): is a tool that provides an analysis with multiple information and metrics. This method integrates the analysis of evolutionary conservation, allelic diversity, variants annotation, functional genomic data, transcription information and causal variants within individual genome sequences. The output provides a numerical score and the higher the raw C score, the more predicted to be deleterious. A score greater or equal 10 indicates that these are the 10% most deleterious substitution. A score of greater or equal 20 indicates the 1% most deleterious and so on[36].
